# Prediction of functional outcome in patients with convulsive status epilepticus: the END-IT score

**DOI:** 10.1186/s13054-016-1221-9

**Published:** 2016-02-25

**Authors:** Qiong Gao, Tang-peng Ou-Yang, Xiao-long Sun, Feng Yang, Chen Wu, Tao Kang, Xiao-gang Kang, Wen Jiang

**Affiliations:** Department of Neurology, Xijing Hospital, Fourth Military Medical University, Xi’an, 710032 PR China

## Abstract

**Background:**

Prediction of the functional outcome for patients with convulsive status epilepticus (CSE) has been a challenge. The aim of this study was to characterize the prognostic factors and functional outcomes of patients after CSE in order to develop a practicable scoring system for outcome prediction.

**Methods:**

We performed a retrospective explorative analysis on consecutive patients diagnosed with CSE between March, 2008 and November, 2014 in a tertiary academic medical center in northwest China. The modified Rankin Scale (mRS) was used to measure the functional outcome at three months post discharge.

**Results:**

A total of 132 CSE patients was included, with a median age of 25.5 years and 60.6 % were male. Three months post discharge, an unfavorable outcome with mRS of 3–6 was seen in 62 (47.0 %) patients, 25 (18.9 %) of whom died. Logistic regression analysis revealed that encephalitis (*p* = 0.029), nonconvulsive SE (*p* = 0.018), diazepam resistance (*p* = 0.005), image abnormalities (unilateral lesions, *p* = 0.027; bilateral lesions or diffuse cerebral edema, *p* < 0.001) and tracheal intubation (*p* = 0.032) were significant independent predictors for unfavorable outcomes. Based on the coefficients in the model, these predictors were assigned a value of 1 point each, with the exception of the image, creating a 6-point scoring system, which we refer to as END-IT, for the outcome prediction of CSE. The area under the receiver operating characteristic curve for the END-IT score was 0.833 and using a cut-off point of 3 produced the highest sum sensitivity (83.9 %) and specificity (68.6 %). Compared with status epilepticus severity score (STESS) and Epidemiology-based Mortality score in SE (EMSE), END-IT score showed better discriminative power and predictive accuracy for the outcome prediction.

**Conclusions:**

We developed an END-IT score with a strong discriminative power for predicting the functional outcome of CSE patients. External prospective validation in different cohorts is needed for END-IT score.

**Electronic supplementary material:**

The online version of this article (doi:10.1186/s13054-016-1221-9) contains supplementary material, which is available to authorized users.

## Background

Convulsive status epilepticus (CSE) is a common, life-threatening neurological disorder [1]. Even with prompt treatment, the mortality rate remains high, ranging from 7.6 to 39 % [2], and more than 10 % of survivors develop neurological and cognitive disabilities [3, 4]. An accurate quantification of CSE severity and a reliable predictor of functional outcomes would be beneficial for clinicians in optimizing individualized patient management and communicating with relatives and other healthcare professionals.

Currently, two scores for SE outcome prediction are available, the Status Epilepticus Severity Score (STESS) [5, 6] and the Epidemiology based Mortality score in SE (EMSE) [7]. The STESS was developed from a retrospective study and was based on four variables at the time of presentation: history of seizures, age, seizure type, and consciousness impairment [6]. The EMSE score was derived from a retrospective, exploratory analysis based on epidemiological data and taking into consideration etiology, age, comorbidity and electroencephalogram (EEG) data [7]. Both of these scores have been primarily used to predict survival vs. death in the hospital setting. However, no scoring system currently exists for the purpose of predicting the functional outcome of patients with CSE once they have been discharged.

Here we performed a retrospective cohort study, on the basis of a prospective registry for SE, with two aims: (1) to identify independent prognostic factors associated with the functional outcome of patients three months after discharge from the hospital setting by analyzing demographic data, clinical features, neuroimages, and the treatment of the disease in the hospital; and (2) to establish a prognostic score by incorporating these variables according to their determined relative contributions to the resulting functional outcome.

## Methods

### Study setting and patients

This retrospective analysis utilized a registry for SE from the neurological intensive care unit (N-ICU) at Xijing Hospital, a tertiary academic medical care institution with 3200-beds in Xi’an, China. All patients with CSE from March 2008 to November 2014 were recruited for this study as long as they were older than 12 years of age. CSE was defined as 30 minutes or more of (1) continuous motor seizure activity or (2) recurrent seizure activity without regaining full consciousness between episodes [8]. Subjects with CSE from cerebral anoxia were excluded due to the high rate of mortality [9]. All patients were followed up for at least three months after discharge from Xijing Hospital. For this, two to three telephone numbers (both mobile and landline) were collected from the patients’ relatives at the time of registry. The present study was approved by the ethics committee of the Xijing Hospital and was carried out in agreement with Chinese laws and the Helsinki declaration relative to patients’ rights.

### SE treatment procedure

All of the CSE patients were treated according to the established hospital protocol, which was based upon published guideline recommendations [10, 11]. Intravenous (IV) diazepam was administered as a first-line antiepileptic drug (AED), followed by IV second-line AEDs if SE persisted (e.g., valproic acid, phenobarbital). If the first- and second-line AEDs failed to treat the patient, third-line treatments with IV anesthetics (e.g., midazolam, propofol) were initiated. Ketamine, isoflurane, and a ketogenic diet were used as a fourth-line therapy.

After admission, each patient received continuous bedside video-EEG monitoring (Solar 2000 N, Solar Electronic Technologies Co., Ltd, Beijing, China) for guiding the AED treatment and detecting the occurrence of nonconvulsive SE (NCSE). NCSE was defined as more than 30 minutes of continuous seizure activity seen on an EEG, with or without subtle motor movements [10, 12]. Upon discharge from the Xijing Hospital, patients were transferred to secondary hospitals where they received rehabilitation treatment.

### Definition of the predictor variables

The following predictor variables with a possible association with the recovery of CSE were chosen for statistical analysis. Three of the chosen variables concerned the demographic and medical history: (1) age; (2) gender; and (3) history of epilepsy, coded as being present or absent.

Two variables were extracted from the clinical examination on N-ICU admission: (1) Glasgow Coma Scale (GCS), dichotomized as a score ranging from 3–8 or ranging from 9–15; and (2) pupillary light reflex, coded as sensitive on both sides, or slow or absence on one side or two sides.

One variable concerned the SE etiology. Given that encephalitis constitutes most of the SE cases, presenting as an acute symptomatic etiology in developing countries, and is related to poor outcome [2, 13–17], the SE etiology was categorized as either encephalitis or non-encephalitis.

Four variables were extracted from the SE treatment: (1) resistance to diazepam, coded as Yes or No; (2) SE duration (time to seizure control), dichotomized as < 2 hrs or ≥ 2 hrs, which is based on previous studies showing that CSE persisting more than 2 hrs was often defined as refractory SE and the patients with CSE exceeding 2 hrs were more likely to have a poor outcome than those with less than 2 hrs [18–20]; (3) drug induced coma; and (4) use of three or more types of intravenous AEDs, both coded as Yes or No.

Three variables concerned comorbidity: (1) complicated with NCSE; (2) tracheal intubation, both coded as Yes or No; and (3) the Charlson’s Comorbidity Index (CCI), classified into three categories: 0 point, 1 to 2 points, 3 points or more [7, 21].

Brain images were also considered as a predictor variable. According to the distribution of responsible lesions on images for SE, we classified brain images into three different categories: no responsible lesion, unilateral responsible lesions, and bilateral responsible lesions or diffuse cerebral edema. We reviewed the first and subsequent study findings of computed tomography (CT) and magnetic resonance imaging (MRI) scans after SE onset and only recorded the most serious findings. The classification of the image was determined by two experienced neurologists unaware of the final outcome, who based their decisions on both pictures and the official radiology reports, and a consensus was reached.

### Definition of outcomes

The clinical outcome was independently assessed three months after discharge from the Xijing Hospital by a trained neurologist, who was blinded to the clinical data, via a telephone interview. The mRS was used to measure the disability of the patients [22]; this scale comprises seven different levels of outcomes ranging from 0 (no symptoms) to 5 (severe disability) and 6 (death). In cases where the patient was unable to complete the interview, the mRS score was determined by interviewing the patient’s caregiver. For the purpose of statistical analysis, we defined the score range of 0–2 (independence) of mRS as a favorable outcome, and the score range of 3–6 (death or dependence with regard to activities of daily living) as an unfavorable outcome.

### Statistical analysis

Continuous variables were expressed as mean ± standard deviation (normally distributed), as medians and interquartile ranges (IQR, not normally distributed), or, if they were categorical variables, they were expressed as counts and percentages. We first conducted univariate comparisons for each outcome using the *χ*^2^ test for the categorical variables and the Mann–Whitney *U* test or Student’s *t*-test for the continuous variables. A backward stepwise multivariate logistic regression was then performed in order to identify independent outcome predictors among those found to have a *p* < 0.05 on the univariate analysis. Points were then assigned to each independent risk factor by dividing its β-coefficient in the model by the lowest β-coefficient and rounding to the nearest integer. Thus, a predictive score was created for each subject by adding the points determined for each factor, with higher scores corresponding to a higher likelihood of an unfavorable outcome for CSE patients. The performance of the predictive score was then evaluated by calculating the area under the receiver operating characteristic curve (ROC). Using the ROC curve, the best cutoff value of the score that was able to predict the primary end point was determined. Predictive accuracy was calculated as the average of sensitivity and specificity. Statistical analyses were performed using the SPSS 18.0 and Matlab 2012a. All tests were two-sided, and a p value of less than 0.05 was considered statistically significant in univariate and multivariate analyses, as was a p value lower than 0.0083 for comparison tests on performances [23].

## Results

**Fig. 1:**
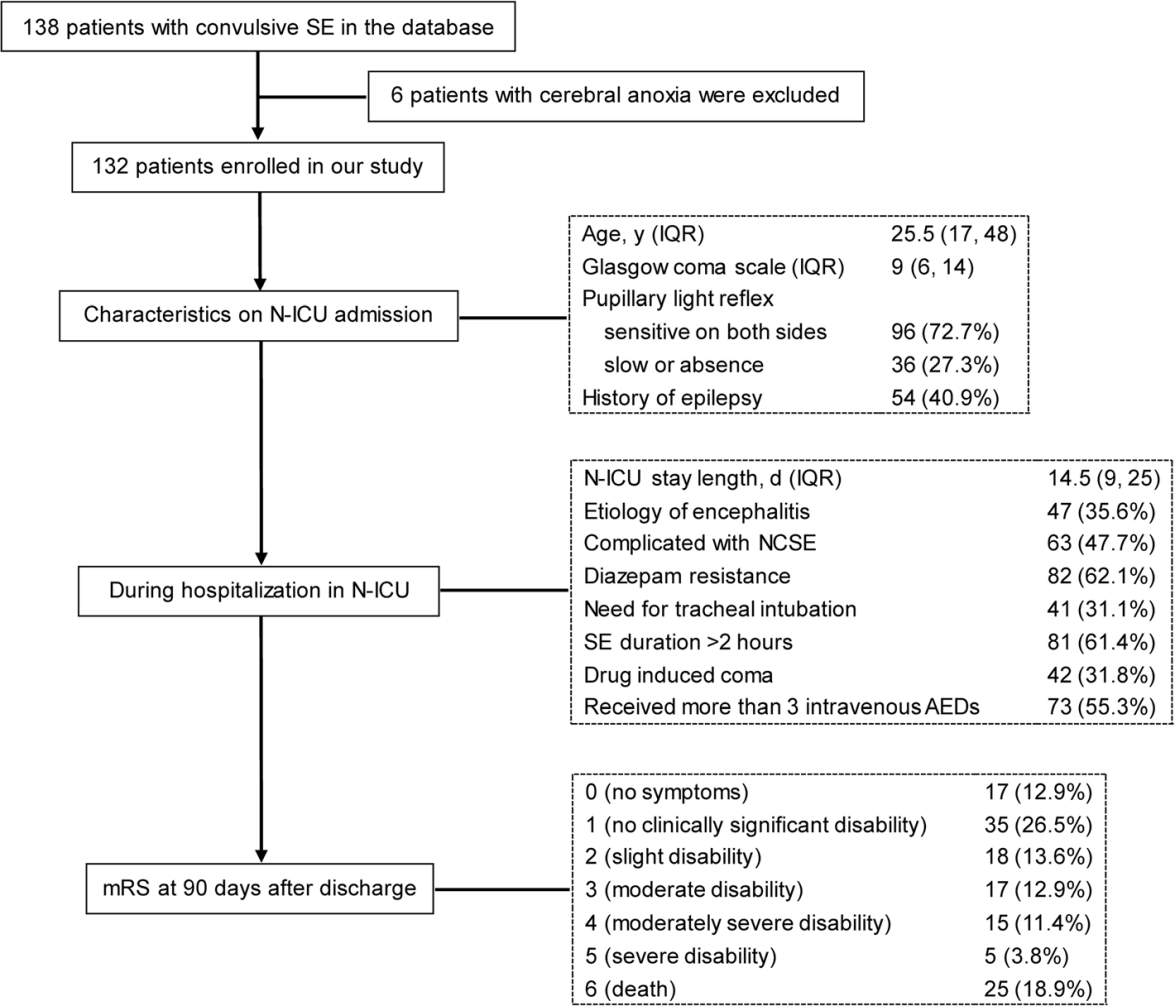
Flow chart describing the characteristics of the study sample

The patient flow chart is shown in Fig. 1. A total of 132 CSE patients were included in the study, each of whom was able to be followed up. The median age of the sample was 25.5 (IQR, 17–48) years and 60.6 % of them were men. The most common cause of CSE in our sample was encephalitis, accounting for 35.6 %, followed by AED low levels/withdrawal (13.6 %) and cerebrovascular diseases (11.4 %), as shown in Table 1. At three months post discharge, 70 (53.0 %) patients presented with a favorable outcome (mRS 0–2), while 62 (47.0 %) patients had an unfavorable outcome (mRS 3–6), among whom 25 (18.9 %) died.

**Table 1:** Causes of convulsive status epilepticus

Etiology	Total No. (%)	mRS (0–2) No. (%)	mRS (3–6) No. (%)
Broad categories			
Acute symptomatic	81 (61.4)	36 (51.4)	45 (72.6)
Remote symptomatic	26 (19.7)	15 (21.4)	11 (17.7)
Progressive symptomatic	5 (3.8)	4 (5.7)	1 (1.6)
Unknown	20 (15.2)	15 (21.4)	5 (8.1)
Individual categories			
Encephalitis	47 (35.6)	15 (21.4)	32 (51.6)
Viral	37 (28.0)	13 (18.6)	24 (38.7)
Tuberculous	4 (3.0)	2 (2.9)	2 (3.2)
Autoimmune	4 (3.0)	0	4 (6.5)
Bacterial	1 (0.8)	0	1 (1.6)
Cryptococcal	1 (0.8)	0	1 (1.6)
AED low levels/withdrawal	18 (13.6)	6 (8.6)	12 (19.4)
Acute cerebrovascular diseases	15 (11.4)	7 (10.0)	8 (12.9)
Metabolism/alcoholism	11 (8.3)	10 (14.3)	1 (1.6)
Acute trauma	2 (1.5)	2 (2.9)	0
Remote trauma	12 (9.1)	9 (12.9)	3 (4.8)
Tumor	3 (2.3)	2 (2.9)	1 (1.6)
Systematic infection	2 (1.5)	2 (2.9)	0
Mitochondrial encephalopathy	1 (0.8)	1 (1.4)	0
Cerebral lupus	1 (0.8)	1 (1.4)	0
Cryptogenic	12 (9.1)	10 (14.3)	2 (3.2)
Two or more causes	8 (6.1)	5 (7.1)	3 (4.8)

*mRS* modified Rankin Scale, *AED* antiepileptic drug

The demographic, clinical, and neuroimage features of the patients during hospitalization in the N-ICU are summarized in Table 2 and Additional file 1. Univariate analysis indicated that age (*p* = 0.075), gender (*p* = 0.387), history of epilepsy (*p* = 0.821), pupillary light reflex on admission (*p* = 0.109), and CCI (*p* = 0.260) did not correlate significantly with the outcome noted at three months post discharge (Table 2). An unfavorable outcome was more likely if the patient had initially presented with a lower GCS score during the time of admission, experienced diazepam resistance and had a longer SE duration, suffered from encephalitis and tracheal intubation, received more than three intravenous AEDs and had a drug induced coma, progressed to NCSE, and displayed abnormal brain images (*p* < 0.05). These variables were then entered into the multivariate logistic regression model, and the results indicated that only encephalitis, NCSE, diazepam resistance, imaging abnormalities, and tracheal intubation were significant independent predictors for an unfavorable outcome (Table 3).

**Table 2:** Univariate analysis

Variable	Total (n = 132)	mRS:0–2 (n = 70)	mRS:3–6 (n = 62)	P
Age, median (IQR), y	25.5 (17.0, 48.0)	24 (17.0, 34.5)	31 (18.8, 54.3)	0.075 (Mann–Whitney *U*)
Gender, male, No. (%)	80 (60.6)	40 (57.1)	40 (64.5)	0.387 (*χ* ^2^)
History of epilepsy, No. (%)	54 (40.9)	28 (40.0)	26 (41.9)	0.821 (*χ* ^2^)
GCS on admission 3–8, No. (%)	54 (40.9)	22 (31.4)	32 (51.6)	0.019 (*χ* ^2^)
Pupillary light reflex on admission, slow or absence, No. (%)	36 (27.3)	15 (21.4)	21 (33.9)	0.109 (*χ* ^2^)
Encephalitis, No. (%)	47 (35.6)	15 (21.4)	32 (51.6)	<0.001 (*χ* ^2^)
Complicated with NCSE, No. (%)	63 (47.7)	23 (32.9)	40 (64.5)	<0.001 (*χ* ^2^)
Diazepam resistance, No. (%)	82 (62.1)	32 (45.7)	50 (80.6)	<0.001 (*χ* ^2^)
Image				0.007 (*χ* ^2^)
unilateral lesions, No. (%)	28 (21.2)	16 (22.9)	12 (19.4)	
bilateral lesions/diffuse cerebral edema, No. (%)	63 (47.7)	25 (35.7)	38 (61.3)	
Tracheal intubation, No. (%)	41 (31.1)	11 (15.7)	30 (48.4)	<0.001 (*χ* ^2^)
Duration >2 h, No. (%)	81 (61.4)	34 (48.6)	47 (75.8)	0.001 (*χ* ^2^)
Drug induced coma, No. (%)	42 (31.8)	15 (21.4)	27 (43.5)	0.006 (*χ* ^2^)
Usage of ≥3 types vAEDs, No. (%)	73 (55.3)	32 (45.7)	41 (66.1)	0.019 (*χ* ^2^)
CCI				0.260 (*χ* ^2^)
1–2	36 (27.3)	17 (24.3)	19 (30.6)	
3 or more	18 (13.6)	5 (7.1)	13 (21.0)	

*mRS* modified Rankin Scale, *IQR* interquartile range, *GCS* Glasgow coma scale, *NCSE* nonconvulsive status epilepticus, *SE* status epilepticus, *vAEDs* intravenous antiepileptic drugs, *CCI* Charlson’s Comorbidity Index

**Table 3:** Multivariable analysis

Variable	Coefficient	Odds ratio (95 % CI)	P	Weighted integer coefficient
Encephalitis	1.105	3.018 (1.119 to 8.141)	0.029	1
NCSE	1.072	2.922 (1.201 to 7.109)	0.018	1
Diazepam resistance	1.361	3.899 (1.519 to 10.007)	0.005	1
Image				
unilateral lesions	1.479	4.386 (1.188 to 16.199)	0.027	1
bilateral lesions/diffuse cerebral edema	1.979	7.236 (2.414 to 21.690)	<0.001	2
Tracheal intubation	1.093	2.982 (1.099 to 8.092)	0.032	1

*NCSE* nonconvulsive status epilepticus

**Fig. 2:**
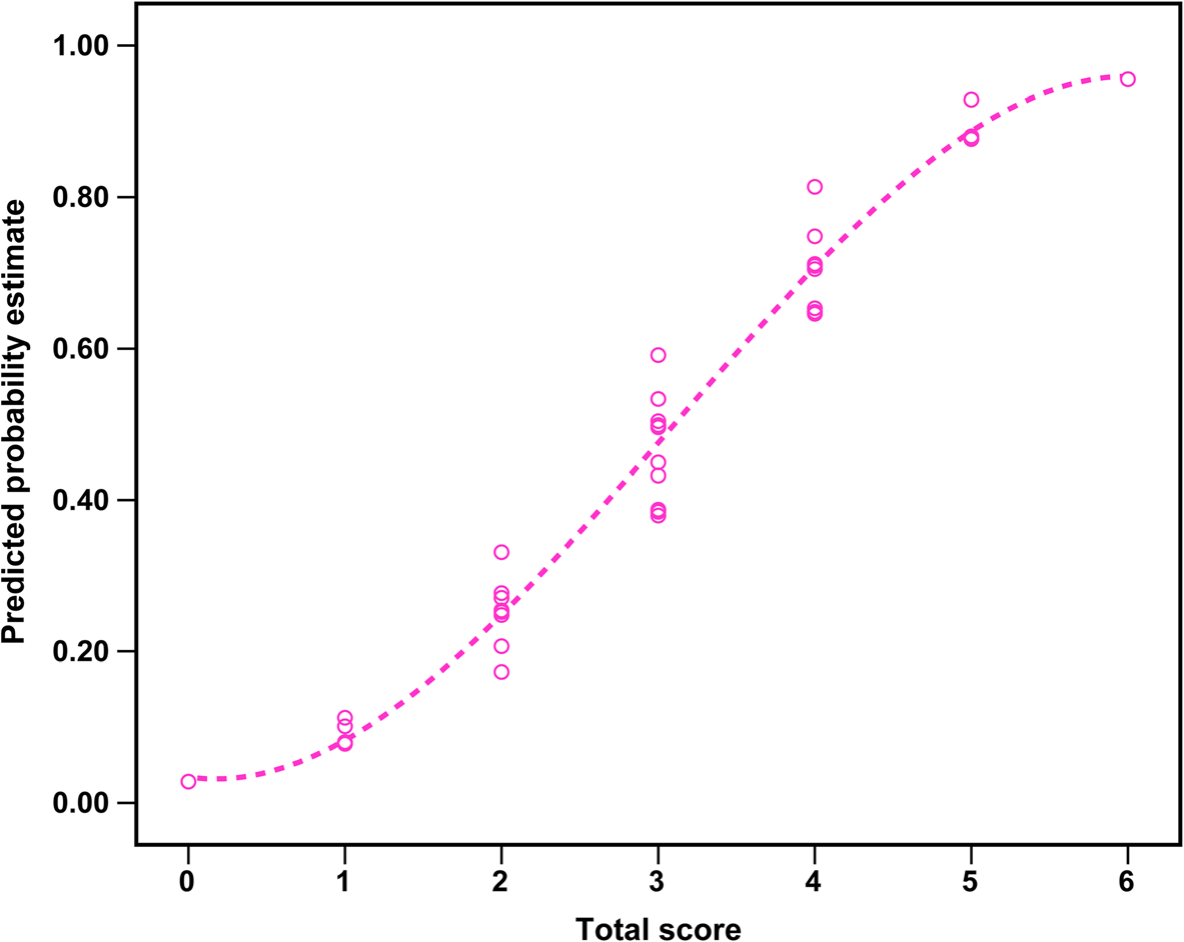
END-IT scores and their corresponding predicted estimates for an unfavorable outcome

In order to establish a straightforward prognostic indicator to be used in clinical practice, we developed a scoring system comprised of the aforementioned five risk factors and named it END-IT, which is an acronym for encephalitis, NCSE, diazepam resistance, image abnormalities and tracheal intubation. Each of the five identified factors was then assigned an integer score based on the magnitude of their determined contribution to adverse events in the multivariate model. The detailed allocation of the scoring points is listed in Table 4. Each of the variables was assigned one point, with the exception of the image, in which unilateral lesions were given one point and bilateral lesions or the presence of diffuse cerebral edema were given two points. The outcome of CSE can be estimated for an individual patient by summing the points of each predictor resulting in a total point score ranging from 0 to 6. The probability of unfavorable outcome increases as the score increases (Fig. 2).

**Table 4:** Point allocation for the END-IT score based on regression coefficients

Relative factor	Categories	Points
Encephalitis	Yes	1
	No	0
NCSE	Yes	1
	No	0
Diazepam resistance	Yes	1
	No	0
Image	bilateral lesions/diffuse cerebral edema	2
	unilateral lesions	1
	no responsible lesion	0
Tracheal intubation	Yes	1
	No	0

*NCSE* nonconvulsive status epilepticus

**Fig. 3:**
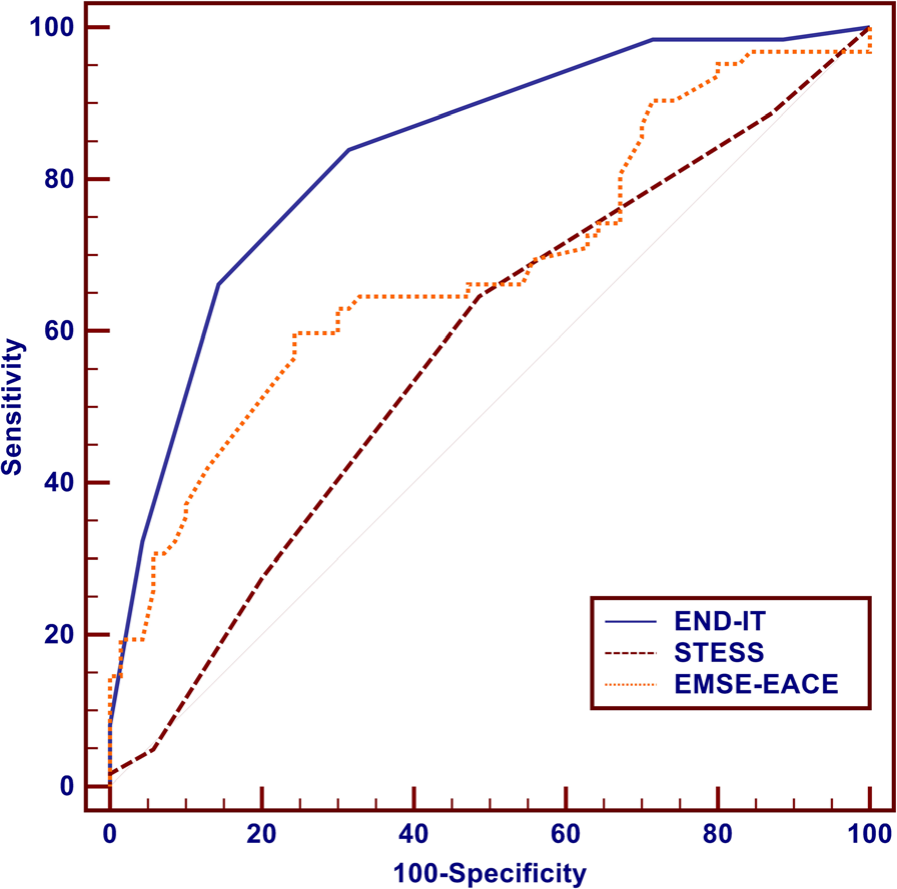
Receiver operating characteristic curve (ROC) for END-IT, Status Epilepticus Severity Score (STESS) and Epidemiology based Mortality score in SE using etiology-age-comorbidity-EEG (EMSE-EACE). END-IT: AUC = 0.833, 95 % CI: 0.766–0.900; STESS: AUC = 0.573, 95 % CI: 0.478–0.668; EMSE-EACE: AUC = 0.683, 95 % CI: 0.590–0.776

The ROC curve for the weighted score was shown to have good discriminative power with an area under the curve (AUC) of 0.833 (95 % CI, 0.758–0.892) (Fig. 3). The validity of the END-IT score in assessing the three-month post discharge outcome in this cohort is summarized in Table 5. The cut-off point of 3 of 6 produced the optimal sum of sensitivity and specificity for the prediction of an unfavorable outcome. Fifty-two (83.9 %) of the 62 patients with an unfavorable outcome had an END-IT score of 3 or greater and 48 (68.6 %) of the 70 patients who presented with a favorable outcome had a score of 0 to 2. Using this cutoff value, the END-IT score had a high sensitivity of 83.9 % with a positive predictive value (PPV) of 70.3 % and a specificity of 68.6 % with a negative predictive value (NPV) of 82.8 % for the prediction of functional outcomes (Table 5). The new prognostic score achieved a predictive accuracy of 76.22 %.

**Table 5:** Performance of the END-IT score

End-it Score	Sensitivity (95 % CI)	Specificity (95 % CI)	PPV (95 % CI)	NPV (95 % CI)
0	100.00 (94.2–100.0)	0.00 (0.0–5.1)	47.0 (38.2–55.8)	
1	98.39 (91.3–100.0)	11.43 (5.1–21.3)	49.6 (40.5–58.8)	88.9 (48.9–99.8)
2	98.39 (91.3–100.0)	28.57 (18.4–40.6)	55.0 (45.2–64.4)	95.2 (75.5–99.9)
3	83.87 (72.3–92.0)	68.57 (56.4–79.1)	70.3 (58.5–80.3)	82.8 (70.4–91.5)
4	66.13 (53.0–77.7)	85.71 (75.3–92.9)	80.4 (66.9–90.2)	74.1 (63.1–83.2)
5	32.26 (20.9–45.3)	95.71 (88.0–99.1)	87.0 (65.8–97.4)	61.5 (51.7–70.6)
6	8.06 (2.7–17.8)	100.00 (94.9–100.0)	100.0 (47.8–100.0)	55.1 (46.0–63.9)

PV was study population specific

*PPV* positive predictive value, *NPV* negative predictive value

**Fig. 4:**
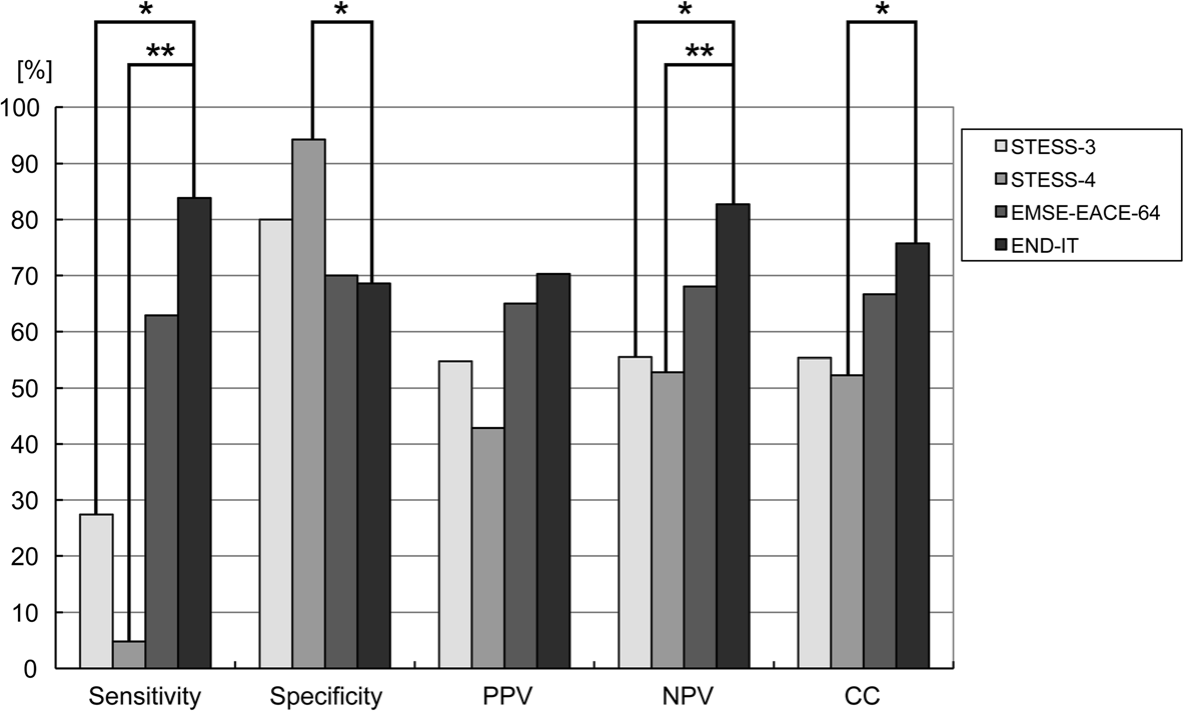
Comparisons of sensitivity, specificity, positive predictive value (PPV) and negative predictive value (NPV), number of correctly classified (CC) patients between STESS-3, STESS-4, EMSE-EACE-64 and END-IT. Sensitivity: *p = 0.0000000028, **p = 0.000000000000046; specificity: *p = 0.00012; NPV: *p = 0.0000030, **p = 0.000000099; correctly classified: *p = 0.00037. Level of significance corrected for multiple testing p < 0.00833. *STESS* status epilepticus severity score, *EMSE* epidemiology based mortality score

STESS and EMSE-score using etiology-age-comorbidity-EEG (EMSE-EACE) were also calculated in this study cohort and compared with the END-IT score in the predictive power of outcome. Figure 3 showed that the END-IT score (AUC = 0.833, accuracy = 76.22 %) had better discriminative power and predictive accuracy than STESS (AUC = 0.573, accuracy = 57.98 %) and EMSE-EACE (AUC = 0.683, accuracy = 67.70 %) in the prediction of functional outcomes. The comparisons of sensitivity, specificity, positive (PPV) and negative (NPV) predictive value and number of correctly classified (CC) patients between STESS, EMSE-EACE and END-IT are shown in Fig. 4. END-IT score was significantly superior to STESS with cutoff levels 3 and 4 (STESS-3: 3 or more points indicate bad outcome, i.e., death; STESS-4: 4 or more points indicate bad outcome, i.e., death): NPV of END-IT 82.76 %, STESS-3 55.45 % (p = 0.0000030), and STESS-4 52.80 % (p = 0.000000099); CC of END-IT 75.76 %, STESS-4 52.27 % (p = 0.00037). Compared with EMSE-EACE with cutoff 64, END-IT score showed a better tendency in NPV and CC but there was no statistically significant difference between them.

## Discussion

A straightforward prognostic scoring system was developed in this study in order to determine the functional outcome of patients with CSE. To make full use of the available clinical data and to systematically consider the progression of SE, our score was initially designed to incorporate the demographics, clinical characteristics, and treatment responses, as well as the available neuroimages. The statistical analysis showed that encephalitis, NCSE, diazepam resistance, image abnormalities and tracheal intubation were significant independent predictors of functional outcome at three months post discharge from the hospital. Using these predictors and their regression coefficients in the model, we developed a simple six-point score, termed END-IT, in order to predict the probability of an unfavorable outcome in CSE. The END-IT score was shown to have a good predictive accuracy as well as good discriminative power, thus providing families and clinicians with better information regarding the prognosis. As the function outcome was known during the process of score generation, this study is explorative, i.e., hypothesis generating.

Given that unfavorable outcome at three months after discharge could be caused by many other factors independent of SE, e.g., car accidents, myocardial infarction, or trauma, it is necessary to exclude these influences in investigating the functional outcome of CSE. Thus, for those patients with unfavorable outcome, we inquired if other risk factors had been encountered after discharge. However, there was no such case in this study cohort. The age of patients had been previously determined by other studies to be an important risk factor for mortality after SE [5, 24–26]. In this study, we did not find a significant relationship between age and functional outcome. This could be partially explained by the fact that there is no direct relationship between age and the severity of clinical conditions, and the latter has an important influence on patients’ recovery. However, it should be noted that the age distribution in the sample group used in our study is different from previous studies. STESS which was created by Rossetti et al. was based on a SE database of 127 episodes occurring in 107 adult patients, in which more than 30 % of the patients were ≥ 65 years of age [24], whereas the median age of our sample group was 25.5 (IQR, 17–48) years of age, and only 11 of the 132 (8.33 %) patients were over 65 years of age. Given that older patients are less resistant to complications of SE and its treatment, such as pneumonia, this could result in a higher incidence of unfavorable outcomes in older patients. A study with a larger sample size and a stratification analysis is needed to clarify this.

Recent studies have indicated that encephalitis is an acute symptomatic etiology present in most cases of SE and is related to a poor prognostic outcome in developing countries [2, 13–17]. Patients with encephalitis were also determined in this study to constitute 35.6 % of all cases of SE, 68.1 % of whom had a score between 3 and 6 at three months post discharge, indicative of an unfavorable functional outcome, which is consistent with the results from previous studies. Therefore, encephalitis was chosen as an etiology variable in the prediction of the functional outcome for CSE patients, which was proven to be an independent predictor by multivariate logistic regression analysis.

NCSE in the END-IT scoring system refers to subtle SE. Subtle SE, a term used to describe SE with subtle clinical features of myoclonic jerks or nystagmus in association with EEG discharges, is a form of NCSE that develops from CSE if the latter has been treated insufficiently [27, 28]. A considerable percentage of patients (47.7 %) suffering from subtle SE, which might be due to structural or metabolic brain damage or an initial insufficient treatment, were found in our study. Previous studies have shown that the presence of NCSE, once CSE was under control, was associated with significant mortality and poor functional outcome [29, 30]. Similarly, we also found that patients with NCSE had a higher proportion of unfavorable outcomes than those without NCSE.

Impairment of consciousness at the onset of SE can be indicative of extreme severity, and has also been shown to be independently related to death in previous retrospective studies [24, 30, 31]. In this study, we used a GCS score and a pupillary light reflex on N-ICU admission in order to evaluate the level of consciousness of each patient with SE. However, a significant relationship between these two variables and the functional outcome was not found. This may be due to the fact that patients on N-ICU admission have received an intravenous diazepam injection in the ambulance or emergency room, which could affect their level of consciousness.

Refractory SE is defined as the presence of SE either clinically or as determined by electrographic examination despite treatment with adequate doses of an initial benzodiazepine followed by a second acceptable AED [10, 32]. The long-term outcome of 596 cases with refractory SE was analyzed by Ferlisi et al., the results of which indicated that 35 % of the patients had died, 13 % had a severe neurological deficit, and only 35 % recovered to baseline [33]. Recent prospective studies have also shown that refractory SE appeared to be strongly associated with poor outcomes [34, 35]. Considering the controversy surrounding the definition of refractory SE, i.e., the number of AEDs that patients need to have failed and the duration of SE after the initiation of treatment [10], we chose several variables that reflect the features of refractory SE, including diazepam resistance, SE duration, use of three or more types of intravenous AEDs, a drug induced coma, and tracheal intubation, as candidate predictors when performing the statistical analysis. Although these variables were shown to be related to an unfavorable outcome in the univariate analysis, multivariate analysis demonstrated that only tracheal intubation functioned as an independent predictor. This could be explained by the fact that tracheal intubation was a consequence possibly linked to diazepam resistance, a longer SE duration, usage of three or more types of intravenous AEDs, and a drug induced coma. Meanwhile, tracheal intubation implied a high risk of respiratory failure, mechanical ventilation, pneumonia, and a longer ICU stay, which were the primary reasons for the poor outcome observed for refractory SE [36, 37].

Recently, Kilbride et al. investigated the clinical outcomes of 63 patients with prolonged refractory SE and found that a normal neuroimage at the onset of SE was associated with a favorable outcome [38]. In an effort to clarify the role of neuroimaging findings in the prediction of functional outcomes, taking into account the extent of brain damage evident on CT/MRI brain scans, we classified them into three types, in which unilateral responsible lesions and bilateral responsible lesions or the presence of diffuse cerebral edema were all significantly associated with unfavorable outcomes. In light of their contribution in multivariate models, unilateral lesions were assigned 1 point, whereas bilateral lesions or diffuse cerebral edema were each given 2 points in the END-IT scoring system. However, because many patients underwent only one imaging examination during N-ICU hospitalization, our study could not demonstrate if an evolution of imaging findings is predictive of a functional outcome.

The limitations of this study should be noted. First, the score was developed based upon data extracted from a relatively small sample size from a single university tertiary medical center. Thus, the results may not be able to be extrapolated to other settings or populations; hence, further validation in different settings is needed. Second, we collected the functional outcome data at three months after discharge, but not three months after onset of CSE. Since the functional outcome is time dependent, it could be influenced by some other factors, e.g., the discharge habits of the different involved physicians, and shortness of hospital beds for fast discharge. Third, our scoring system did not take into account serological variables, e.g., serum neuron-specific enolase (NSE). Although a few reports have previously shown that NSE may be a promising in vivo marker for brain injury after SE [39–42], its exact role in prognosis of CSE awaits further research. Fourth, we did not examine the role of certain EEG patterns in outcome prediction. Long-term EEG monitoring revealed that some patients often presented with several different kinds of EEG patterns, e.g., burst-suppression and postictal discharges could coexist with difference in sequence, which makes it difficult to interpret the relationship between a specific EEG pattern and a functional outcome. Lastly, we collected the follow-up information on function outcome of patients through telephone interviews but not clinical examination. This might result in some small biases in outcome information, since mRS based on telephone interview could be influenced by some complicating factors.

## Conclusions

In this study, we developed a straightforward and practicable scoring system, termed END-IT, to predict the three-month functional outcome post discharge for CSE patients. The score comprises five in-hospital variables: encephalitis, NCSE, diazepam resistance, image, and tracheal intubation, and showed good predictive accuracy and discrimination power. However, it should be remembered that the END-IT score simply predicts the probability of functional outcome, with no judgment being made as to whether the patients continue to receive intensive treatment or not. Future studies are required for further validation and improvements upon this scoring system.

## Key messages

- Forty-seven percent of patients with convulsive status epilepticus lasting more than 30 minutes suffered an unfavorable outcome.
- Encephalitis, NCSE, diazepam resistance, image abnormalities and tracheal intubation were independent predictors for unfavorable outcome.
- Based on these five independent predictors and their coefficients in the logistic regression model, we established a 6-point scoring system, termed END-IT.
- The END-IT score showed good discriminative power as well as predictive accuracy.

## Additional file

Additional file 1:
**Baseline characteristics of 132 patients.** (DOC 42 kb)

## Abbreviations

AED: Antiepileptic drug
AUC: Area under the curve
CC: Correctly classified
CCI: Charlson’s Comorbidity Index
CSE: Convulsive status epilepticus
CT: Computed tomography
EACE: Etiology-age-comorbidity-EEG
EEG: electroencephalogram
EMSE: Epidemiology based mortality score
GCS: Glasgow Coma Scale
IQR: Interquartile range
IV: Intravenous
MRI: Magnetic resonance imaging
mRS: Modified Rankin Scale
NCSE: Nonconvulsive status epilepticus
N-ICU: Neurological intensive care unit
NPV: Negative predictive value
NSE: Neuron-specific enolase
PPV: Positive predictive value
ROC: Receiver operating characteristic curve
STESS: Status epilepticus severity score
