# Supplementary material for: Prediction of functional outcome in patients with convulsive status epilepticus: the END-IT score
Source: Crit Care. 2016 Feb 25;20:46. doi: 10.1186/s13054-016-1221-9 (PMC4768332; doi:10.1186/s13054-016-1221-9)
Supplement: Additional file 1: — Baseline characteristics of 132 patients. (DOC 42 kb) [file 13054_2016_1221_MOESM1_ESM.doc]

**Additional file 1** Baseline characteristics of 132 patients

| **Variable** | **Total, No. (%)** | **mRS (0-2), No. (%)** | **mRS (3-6), No. (%)** |
| --- | --- | --- | --- |
| SE onset to endpoint (IQR), d | 108  (103.0, 123.8) | 107  (103.8,120.8) | 111  (100.0, 129.8) |
| Duration of EEG monitoring (IQR), h | 72 (48.0, 77.8) | 72 (24.0, 72.0) | 72 (72.0, 90.3) |
| Convulsive SE |  |  |  |
| Generalized convulsive, No. (%) | 73 (55.3) | 40 (57.1) | 33 (53.2) |
| Focal onset evolving into bilateral convulsive SE, No. (%) | 59 (44.7) | 30 (42.9) | 29 (46.8) |
| Acute or Remote lesions |  |  |  |
| Acute, No. (%) | 57 (43.2) | 25 (35.7) | 32 (51.6) |
| Remote, No. (%) | 34 (25.8) | 16 (22.9) | 18 (29.0) |
| Types of imaging examinations |  |  |  |
| CT only, No. (%) | 19 (14.4) | 7 (10.0) | 12 (19.4) |
| MRI only, No. (%) | 23 (17.4) | 12 (17.1) | 11 (17.7) |
| CT and MRI, No. (%) | 90 (68.2) | 51 (72.9) | 39 (62.9) |
| Cause of tracheal intubation |  |  |  |
| Respiratory failure, No. (%) | 10 (7.6) | 2 (2.9) | 8 (12.9) |
| Treatment of SE, No. (%) | 31 (23.5) | 9 (12.8) | 22 (35.5) |
| STESS ≥ 3 points, No. (%) | 31 (23.5) | 14 (20.0) | 17 (27.4) |
| STESS ≥ 4 points, No. (%) | 7 (5.3) | 4 (5.7) | 3 (4.8) |
| EMSE-EACE ≥ 64 points, No. (%) | 60 (45.5) | 21 (30.0) | 39 (62.9) |

Abbreviations: IQR, Interquartile range; NCSE, nonconvulsive status epilepticus; SE, status epilepticus; STESS, Status Epilepticus Severity Score; EMSE-EACE, Epidemiology based Mortality score in SE- aetiology-age-comorbidity-EEG.
